# Inflammation-associated rapidly progressive coronary artery disease: a case report

**DOI:** 10.1093/ehjcr/ytag177

**Published:** 2026-03-10

**Authors:** Liang Wang, Lun Wang, Yifan Liu, Lidan Zhao, Zhenyu Liu

**Affiliations:** Department of Cardiology, Peking Union Medical College Hospital, Chinese Academy of Medical Sciences & Peking Union Medical College, No. 1 Shuaifuyuan, Dongcheng District, Beijing 100730, China; Department of Cardiology, Peking Union Medical College Hospital, Chinese Academy of Medical Sciences & Peking Union Medical College, No. 1 Shuaifuyuan, Dongcheng District, Beijing 100730, China; Department of Cardiology, Peking Union Medical College Hospital, Chinese Academy of Medical Sciences & Peking Union Medical College, No. 1 Shuaifuyuan, Dongcheng District, Beijing 100730, China; Department of Rheumatology and Clinical Immunology, Peking Union Medical College Hospital, Chinese Academy of Medical Sciences & Peking Union Medical College, No.1 Shuaifuyuan, Dongcheng District, Beijing 100730, China; Department of Cardiology, Peking Union Medical College Hospital, Chinese Academy of Medical Sciences & Peking Union Medical College, No. 1 Shuaifuyuan, Dongcheng District, Beijing 100730, China

**Keywords:** Case report, Coronary artery disease, Coronary restenosis, Disease progression, Immunosuppressive therapy, Inflammation, Percutaneous coronary intervention

## Abstract

**Background:**

Inflammation plays important roles in the pathogenesis of both *de novo* and restenotic lesions in coronary artery disease (CAD).

**Case summary:**

This patient was a middle-aged female who had few risk factors for atherosclerotic CAD (AS-CAD) but prior history of psoriasis and elevated erythrocyte sedimentation rate. The patient experienced four ischaemia-driven hospitalizations and/or percutaneous coronary interventions (PCIs) within 18 months due to new-onset or worsened coronary *de novo* and/or restenotic lesions, especially recurrent restenosis at the same vessel segment, despite optimal PCI and strict secondary prevention for AS-CAD. The patient received: (i) ischaemia-driven (least-necessary) and restricted (least-invasive) PCI, which relieved severe anginal symptoms and (ii) immunosuppressive therapy, which was associated with delayed progression and even partial regression of the coronary lesions, as well as reduced need for further ischaemia-driven hospitalization and/or revascularization during a 40-month follow-up.

**Discussion:**

We propose to use inflammation-associated rapidly progressive CAD (IR-CAD) to define the disease entity of this patient. Both systemic inflammation and local vascular inflammation secondary to local vascular mechanical injury, such as that induced by PCI *per se*, might contribute to the pathogenesis of IR-CAD. Immunosuppressive therapy (to control inflammation) together with ischaemia-driven and restricted PCI (to relieve severe myocardial ischaemia and avoid unnecessary vascular mechanical injury) might be crucial to the treatment of IR-CAD.

Learning points

- Inflammation-associated rapidly progressive coronary artery disease (IR-CAD) is characterized by manifestations of inflammation and rapid progression of coronary *de novo* and/or restenotic lesions.
- Both systemic inflammation and local vascular inflammation secondary to local vascular mechanical injury might involve in the pathogenesis of IR-CAD.
- Immunosuppressive therapy together with ischaemia-driven and restricted percutaneous coronary intervention might be crucial to the treatment of IR-CAD.

## Introduction

Inflammation plays important roles in the pathogenesis of both *de novo* (atherosclerosis)^1^ and restenotic (neointimal hyperplasia) lesions in coronary artery disease (CAD).^2^ This case report described a patient with inflammation-associated rapidly progressive CAD (IR-CAD), which highlighted the importance of inflammation in the pathogenesis as well as immunosuppressive therapy (IST) and avoidance of vascular mechanical injury in the treatment of IR-CAD.

## Summary figure

**Figure:**
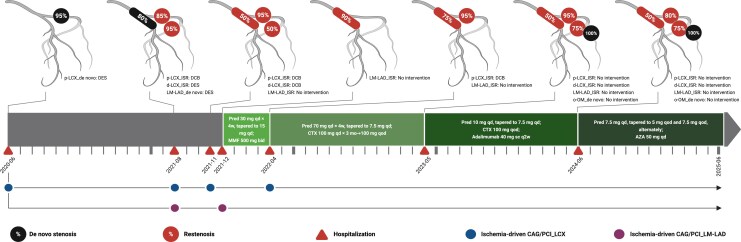
Clinical course, angiographic results and treatment.

## Case presentation

A 47-year-old woman was admitted to our hospital due to recurrent chest pain for 18 months.

**Figure 1:**
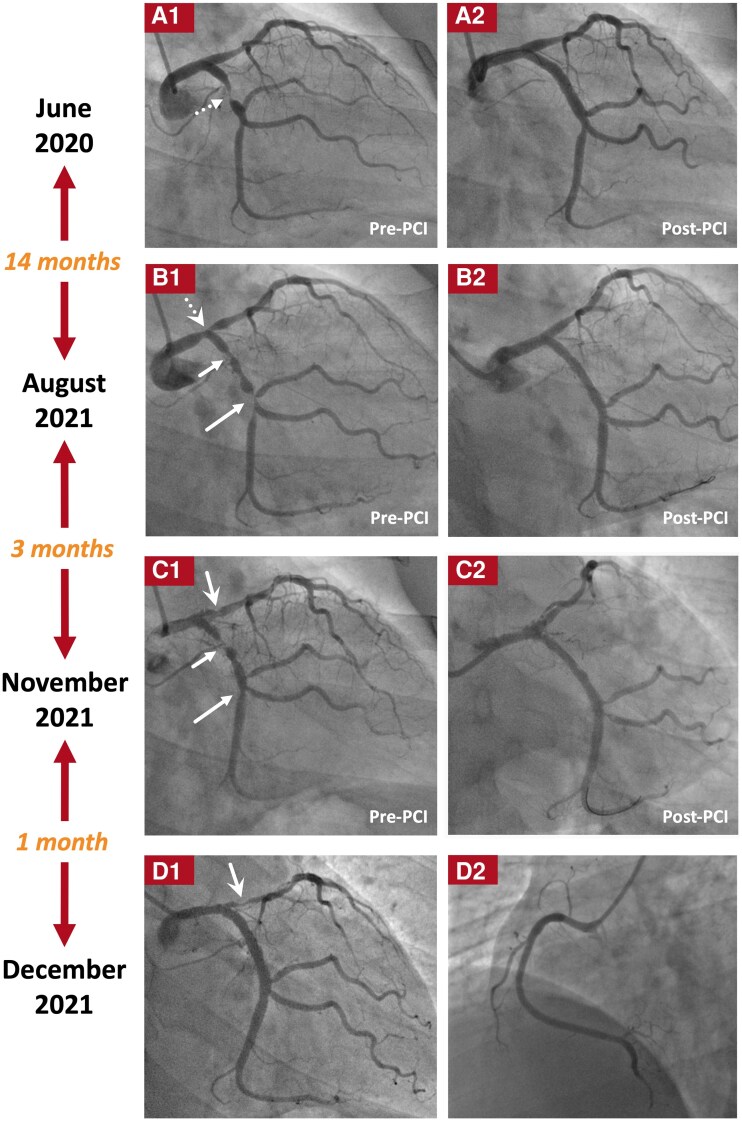
Coronary angiograms before initiation of IST. The coronary angiograms before and after each PCI during the first (June 2020) (A1 and A2), the second (August 2021) (B1 and B2), and the third (November 2021) (C1 and C2) hospitalizations (all ischaemia driven) at the local hospital, as well as the coronary angiograms during the first hospitalization (ischaemia driven) in our hospital (December 2021) (D1 and D2) are shown. Before the initiation of IST, coronary *de novo* stenosis and/or restenosis occurred after each PCI (B1, C1, D1) with progressively shorter time intervals between two consecutive ischaemia-driven hospitalizations and/or PCIs (14 months [June 2020 to August 2021] → 3 months [August 2021 to November 2021] → 1 month [November 2021 to December 2021]), as well as increasingly more vessel segments affected (proximal LCX [short dashed arrow] [A1] → proximal LCX [short solid arrows], distal LCX [long solid arrows], and distal LM-proximal LAD [dashed and solid swallowtail arrows] [B1, C1, D1]). The RCA remained intact before the initiation of IST (D2). Different types of arrows are used to point out the *de novo* lesion in the proximal LCX (short dashed arrow), the restenotic lesions in the proximal LCX (short solid arrows) and the distal LCX (long solid arrows), as well as the *de novo* (dashed swallowtail arrow) and restenotic (solid swallowtail arrows) lesions in the distal LM-proximal LAD. IST, immunosuppressive therapy; LAD, left anterior descending artery; LCX, left circumflex artery; LM, left main trunk; PCI, percutaneous coronary intervention; RCA, right coronary artery.

Between June 2020 and December 2021, the patient experienced four hospitalizations within 18 months due to chest pain, including three hospitalizations in a local hospital (June 2020, August 2021, November 2021, respectively), and the index hospitalization in our hospital (December 2021) (Summary figure). The time intervals between two consecutive hospitalizations were progressively shorter (14 months → 3 months → 1 month). Coronary angiography (CAG) showed new-onset or worsened *de novo* stenosis and/or restenosis during each hospitalization (*Figure 1*). Percutaneous coronary interventions (PCIs) were performed with new-generation drug-eluting stents (DESs) and/or drug-coated balloons (DCBs) during the first three hospitalizations. However, *de novo* stenosis and/or restenosis occurred after each PCI with increasingly more vessel segments affected (proximal left circumflex artery [LCX] → proximal LCX, distal LCX, and distal left main trunk [LM]-proximal left anterior descending artery [LAD]).

The patient was a nonsmoker. She had been diagnosed with mild psoriasis for 7 years, which had been clinically quiescent for 5 years after 2 years of treatment. During the index hospitalization, she had normal and symmetrical blood pressure, and a body mass index of 32.3 kg/m^2^. Low-density lipoprotein cholesterol (1.47 mmol/L) and glycated haemoglobin (5.5%) were well controlled, but erythrocyte sedimentation rate (ESR) (63 mm/h) was elevated (*Table 1*). Other inflammatory markers, autoantibodies, and cardiac biomarkers were negative. Electrocardiography and echocardiography revealed no obvious abnormality. No revascularization was performed. Instead, IST (oral prednisone and mycophenolate mofetil) was given in addition to strict secondary prevention (Summary figure, *Table 2*). Her symptoms improved within a few days.

**Table 1:** Examinations

Time of hospitalizations		June	August	November	December	April	May	June
		2020	2021	2021	2021	2022	2023	2024
Laboratory tests	ESR (< 20 mm/h)	NA	NA	NA	63	11	12	6
	hs-CRP (< 2 mg/L)	NA	NA	NA	1.89	0.61	0.82	< 0.50
	LDL-C (< 1.4 mmol/L)	3.26	NA	NA	1.47	1.46	1.24	1.30
	HbA1c (< 6.3%)	6.3	NA	NA	5.5	6.9	7.5	7.1
Body mass index (kg/m^2^)		NA	NA	NA	32.3	33.4	34.4	32.6
LVEF on echocardiography (%)		65	NA	NA	68	66	62	61
Maximal plaque thickness of left carotid artery on ultrasound (mm)		NA	NA	NA	2	1.6	1.8	1.9

ESR, erythrocyte sedimentation rate; HbA1c, glycated haemoglobin; hs-CRP, high-sensitivity C-reactive protein; LDL-C, low-density lipoprotein cholesterol; LVEF, left ventricular ejection fraction; NA, not applicable.

**Table 2:** Medical treatment

Time of hospitalizations	June	August	November	December	April	May	June
	2020	2021	2021	2021	2022	2023	2024
Aspirin	100 mg qd	100 mg qd	100 mg qd	100 mg qd	100 mg qd	100 mg qd	None
P2Y12 inhibitor	Clopidogrel	Ticagrelor	Ticagrelor	Ticagrelor	Ticagrelor	Ticagrelor	Ticagrelor
	75 mg qd	90 mg bid	90 mg bid	90 mg bid	90 mg bid	90 mg bid	90 mg bid
Statin	Atorvastatin	Atorvastatin	Atorvastatin	Atorvastatin	Rosuvastatin	Rosuvastatin	Rosuvastatin
	20 mg qn	20 mg qn	20 mg qn	20 mg qn	10 mg qn	10 mg qn	10 mg qn
Ezetimibe	None	None	None	10 mg qd	10 mg qd	10 mg qd	10 mg qd
Dapagliflozin	None	None	None	None	None	10 mg qd	10 mg qd
Semaglutide	None	None	None	None	None	0.75 mg qw^a^	None
Metoprolol Tartrate	12.5 mg q12h	12.5 mg q12h	12.5 mg q12h	37.5 mg q12h	87.5 mg q12h	87.5 mg q12h	87.5 mg q12h
Nicorandil	None	None	None	5 mg tid	5 mg tid	5 mg tid	5 mg tid
Immunosuppressive therapy	None	None	None	Prednisone + MMF	Prednisone + CTX	Prednisone + CTX + adalimumab	Prednisone + AZA

AZA, azathioprine; CTX, cyclophosphamide; MMF, mycophenolate mofetil.

^a^The patient discontinued semaglutide due to personal reasons in February 2024.

**Figure 2:**
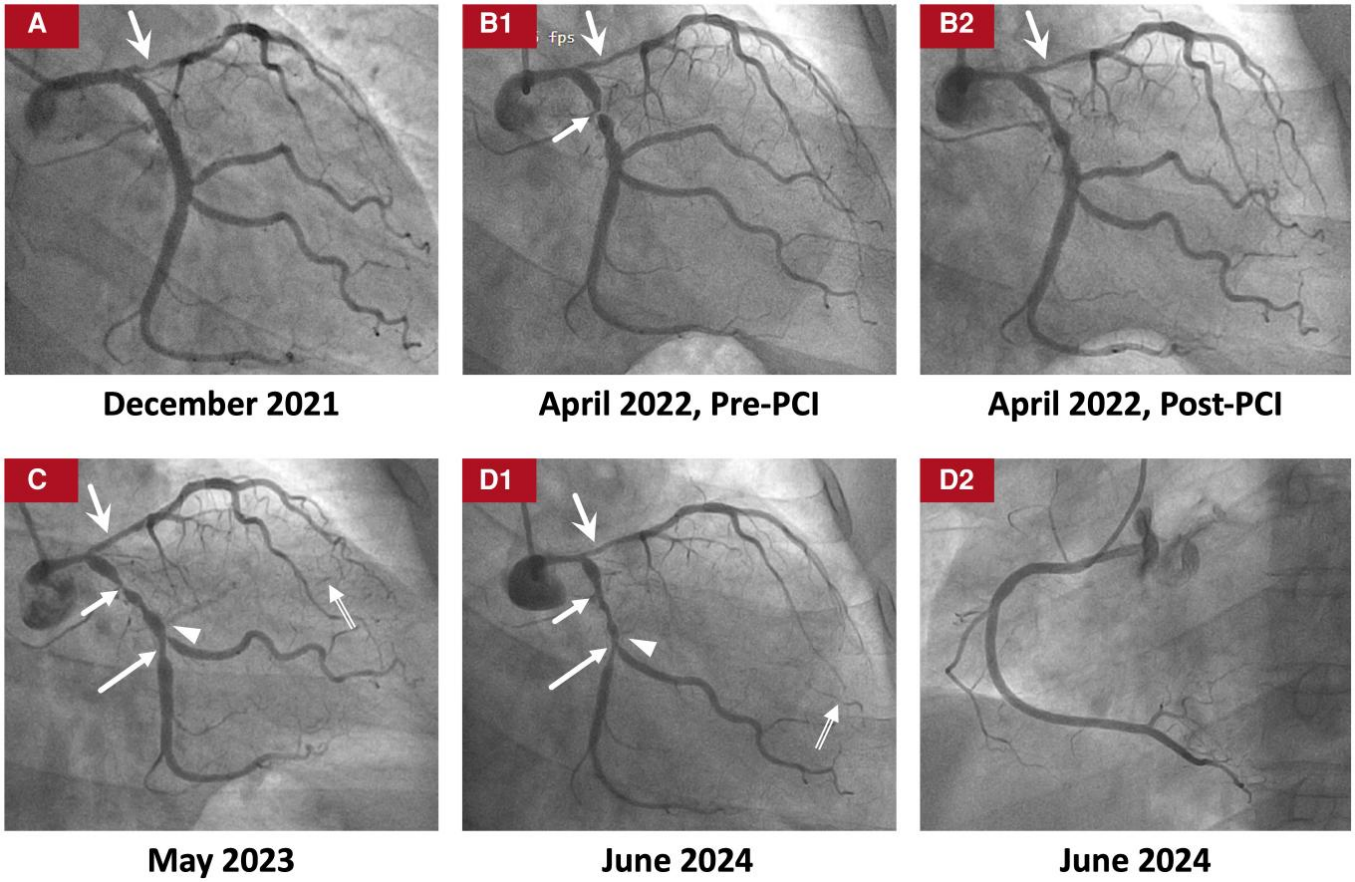
Coronary angiograms immediately before and after initiation of IST. The coronary angiograms immediately before the initiation of IST during the first hospitalization (ischaemia driven, December 2021) (*A*), as well as those after the initiation of IST during the second (ischaemia driven, April 2022) (B1 and B2), the third (planned, May 2023) (*C*), and the fourth (planned, June 2024) (D1 and D2) hospitalizations in our hospital are shown. After the initiation of IST, (1) the lumen diameter in the distal LM-proximal LAD (solid swallowtail arrows) continuously improved (90% → 75% → 50% diameter stenosis) (Figure A-D1) and (2) although restenosis did recur in the proximal LCX (short solid arrows) even after more than 1 year of treatment with IST (*C*), the lumen diameter in the proximal LCX also slightly improved (95% → 80% diameter stenosis) after more than 2 years of treatment with more intensified IST (D1). The RCA remained intact after the initiation of IST (D2). Different types of arrows are used to point out the restenotic lesions in the proximal LCX (short solid arrows), distal LCX (long solid arrows), and distal LM-proximal LAD (solid swallowtail arrows), as well as the *de novo* total occlusion in the ostium of the OM1 (arrowheads), and the collateral circulation from the LCA to the OM1 (double-line arrows). IST, immunosuppressive therapy; LAD, left anterior descending artery; LCA, left coronary artery; LCX, left circumflex artery; LM, left main trunk; OM1, the first obtuse marginal branch; PCI, percutaneous coronary intervention; RCA, right coronary artery.

**Figure 3:**
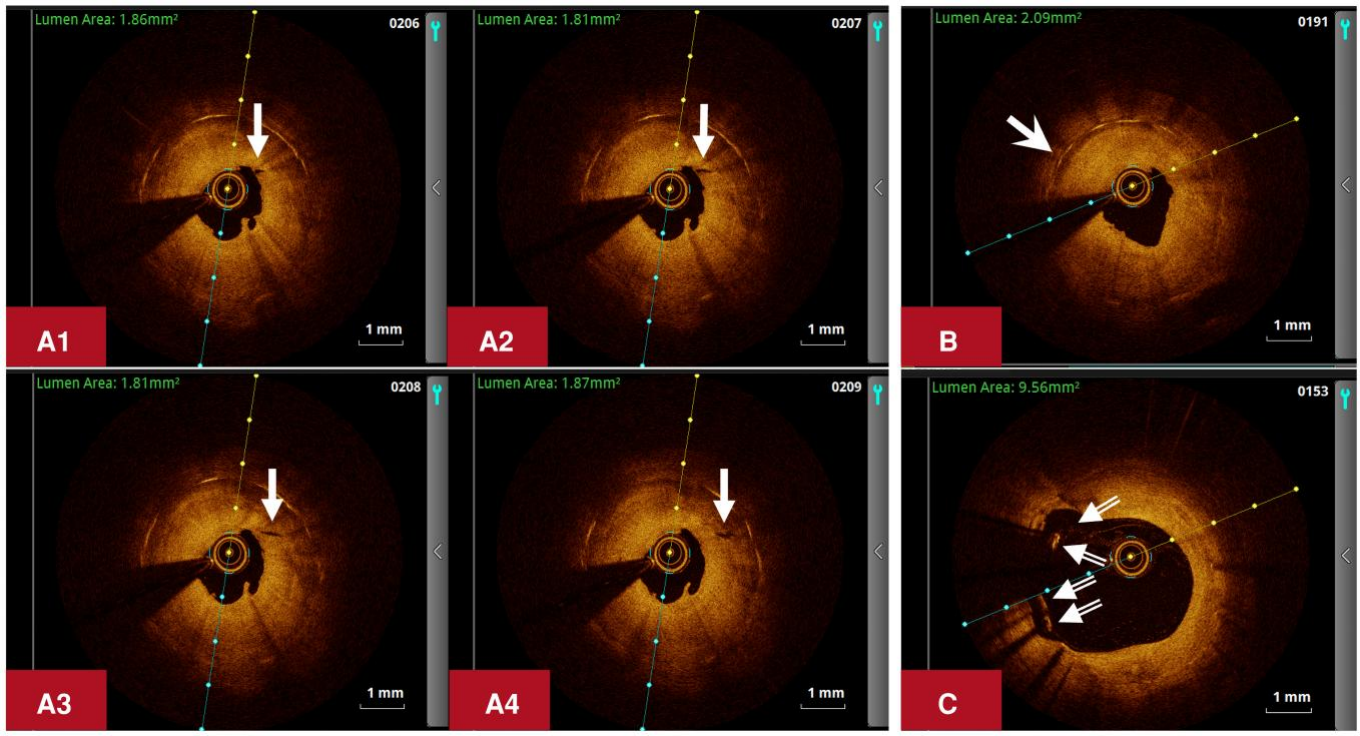
OCT images in the proximal LCX. OCT was performed in the LCX after predilating the restenotic lesion (95% diameter stenosis) in the proximal LCX with a plain balloon (2.0 × 15 mm) during the second hospitalization (ischaemia driven, April 2022) in our hospital. The OCT images revealed significant concentric neointimal thickening with multiple microchannels communicating with the vessel lumen (A1-A4, solid arrows), peri-strut low-intensity area (*B*, solid swallowtail arrow), and stent malapposition (*C*, double-line arrows) in the proximal LCX. LCX, left circumflex artery; OCT, optical coherence tomography.

Four months after discharge, the patient was admitted to our hospital due to chest pain for the second time in April 2022. ESR was well controlled (*Table 1*). CAG demonstrated improvement of the lumen diameter in the distal LM-proximal LAD, but 95% in-stent restenosis in the proximal LCX (*Figure 2B1*). After predilation with a plain balloon (2.0 mm × 15 mm), optical coherence tomography (OCT) performed in the LCX revealed significant concentric neointimal thickening with multiple microchannels communicating with the vessel lumen, peri-strut low-intensity area, and stent malapposition only in the vessel segment covered by the proximal LCX stent (*Figure 3*). The lesion was treated with an undersized DCB (2.5 mm × 25 mm) after predilation with an undersized scoring balloon (2.5 mm × 13 mm) (*Figure 2B2*).

After discharge, the patient received more intensified IST (oral prednisone and cyclophosphamide, and subcutaneous adalimumab only during the second year after discharge) (Summary figure). Up to August 2025, no unplanned hospitalizations occurred during a 40-month follow-up. Annually planned CAGs (May 2023 and June 2024) showed: (i) the lumen diameter in the distal LM-proximal LAD continuously improved (90% → 75% → 50% diameter stenosis) (*Figure 2A-2D1*) and (ii) although restenosis recurred in the proximal LCX after more than 1 year of treatment with IST (*Figure 2C*), the lumen diameter in the proximal LCX slightly improved (95% → 80% diameter stenosis) after more than 2 years of treatment with more intensified IST (*Figure 2D1*). The RCA remained intact during the entire course (*Figure 1D2* and *Figure 2D2*).

## Discussion

The clinical features of this patient are significantly different from those of typical atherosclerotic CAD (AS-CAD) patients, but quite similar to those of a recently reported case with inflammation-associated CAD (I-CAD),^3^ including (i) early-onset CAD despite few traditional risk factors for AS-CAD, (ii) rapidly progressive coronary *de novo* stenosis and restenosis despite optimal revascularization and strict secondary prevention, (iii) manifestations of inflammation (history of psoriasis and elevated ESR) and (iv) positive treatment response to IST both clinically and angiographically. Considering the above clinical features of this patient and the important roles of inflammation in the pathogenesis of coronary *de novo* stenosis^1^ and restenosis,^2^ we speculate that uncontrolled systemic inflammation might contribute to the early development and rapid progression of the coronary lesions in this patient.

The distributions of the *de novo* and restenotic lesions in the coronary vasculature of this patient were uneven. (i) The *de novo* lesions usually located in the proximal segments of the coronary vessels, which might be related to the vascular mechanical injury caused by the shear stress of blood flow and/or the interventional devices advancing and/or withdrawing through the proximal vessel segments. (ii) Rapidly progressive, recurrent restenosis usually occurred at the vessel segments receiving multiple interventional treatment, suggesting that local vascular mechanical injury induced by interventional treatment *per se* might be associated with the rapid development of restenosis.^4^ In summary, local vascular mechanical injury, which leads to local vascular inflammation, might also participate in the pathogenesis of the *de novo* and restenotic lesions in this patient in addition to the background systemic inflammation.

This patient demonstrated not only the disease characteristics of I-CAD (manifestations of inﬂammation and progressive CAD) but also more rapid and accelerating disease progression, more severe anginal symptoms, and more urgent need for revascularization compared to the previous case.^3^ Therefore, we propose to use the concept of IR-CAD to describe the disease entity of this patient, which is defined as I-CAD with rapid progression, i.e. ischaemia-driven hospitalization and/or revascularization due to new-onset or worsened coronary *de novo* and/or restenotic lesions occurring within 6 months of the latest CAG and/or revascularization.

The rationale for using IST in the management of I-CAD was elaborated in the previous case report, in which IST was associated with regression of the *de novo* and the traumatic lesions but not the two restenotic lesions with total occlusion.^3^ In contrast to the previous case, IST was associated with partial regression of the restenotic lesions in both the distal LM-proximal LAD and the proximal LCX in this patient. Taken together, IST might be effective in controlling the progression of all three types of lesions in I-CAD/IR-CAD.

As mentioned, revascularization *per se* might accelerate the progression of I-CAD, which should be avoided as much as possible or be performed only after both systemic inflammation and local vascular inflammation have been adequately controlled. However, revascularization is usually unavoidable if I-CAD patients have developed severe anginal symptoms before inflammation can be adequately controlled by IST, especially for IR-CAD patients with rapid disease progression and tight vessel stenosis. In this case, a strategy of ischaemia-driven (least-necessary) and restricted (least-invasive) PCI might be the treatment choice for IR-CAD patients to alleviate anginal symptoms and to gain enough time for IST to control inflammation, while at the same time, to avoid unnecessary vascular mechanical injury as much as possible. In technical aspects, under-sized cutting or scoring balloons^5–7^ (for lesion pretreatment) and under-sized DCBs (as final treatment) might be the appropriate interventional devices for restricted PCI (as in this patient), which might not only reduce excessive vascular mechanical injury but also avoid leaving metal and/or polymer in the vessel wall compared to full-sized DESs.^8^

Regarding the source of systemic inflammation in this patient, several potential aetiologies should be considered. First, psoriasis, a systemic inflammatory disease, is associated with increased risk of CAD.^9^ Second, both atherosclerosis and neoatherosclerosis are inflammatory processes, promoting the development of coronary *de novo* stenosis and in-stent restenosis, respectively.^10,11^ Third, obesity may lead to chronic low-grade systemic inflammation, driving obesity-related cardiometabolic disease, including CAD.^12–14^

Intravascular imaging techniques may provide imaging findings suggestive of inflammation in the arterial vessel wall.^8,15–17^ As in this patient, OCT demonstrated highly proliferative characteristics of the restenotic lesions in the LCX, suggesting the existence of over-activated inflammatory response to the vascular mechanical injury associated with PCI. However, most of the patients with I-CAD/IR-CAD, including this patient, did not undergo intravascular imaging examinations prior to their first PCI, which precludes the potential opportunity to demonstrate the inflammatory nature of their original coronary lesions.

In conclusion, in patients with manifestations of inflammation and rapidly progressive CAD, the diagnosis of IR-CAD should be considered. Both systemic inflammation and local vascular inflammation secondary to local vascular mechanical injury might involve in the pathogenesis of IR-CAD. IST together with ischaemia-driven and restricted PCI might be crucial to the treatment of IR-CAD.

## Data Availability

The data underlying this article will be shared on reasonable request to the corresponding authors.

## Lead author biography

Dr. Zhenyu Liu, M.D., is the director of the Department of Cardiology, Peking Union Medical College Hospital, Beijing, China. He is an interventional cardiologist focusing on percutaneous coronary intervention for more than 20 years. His research interest is inflammation-associated coronary artery disease.

## Notes

**Consent:** The authors confirm that written consent for submission and publication of this case report has been obtained from the patient in line with the COPE guidelines.
